# Gut Mycobiota Dysbiosis in People Living with HIV/AIDS: Insights from an Argentine Cohort with Severe Immunosuppression

**DOI:** 10.3390/jof12050306

**Published:** 2026-04-23

**Authors:** Cristian Javier Mena, Néstor Denis Portela, Agostina Salusso, Andrés Barnes, César Collino, Silvia Guadalupe Carrizo, Davor Martinovic, Mariel A. Almeida, Lizet Luque Aguada, Lorena Guasconi, Martín Gustavo Theumer, Laura Cervi, Susana Alicia Pesoa, Laura Silvina Chiapello

**Affiliations:** 1 Departamento de Bioquímica Clínica, Facultad de Ciencias Químicas, Universidad Nacional de Córdoba, Córdoba X5000HUA, Argentina; cristian.mena@unc.edu.ar (C.J.M.); agosalusso@gmail.com (A.S.); mariel.almeida@mi.unc.edu.ar (M.A.A.); lizet.luque@gmail.com (L.L.A.); lguasconi@unc.edu.ar (L.G.); mgtheumer@unc.edu.ar (M.G.T.); laura.cervi@unc.edu.ar (L.C.); 2 Centro de Investigaciones en Bioquímica Clínica e Inmunología (CIBICI), Consejo Nacional de Investigaciones Científicas y Técnicas (CONICET), Ciudad Universitaria, Córdoba X5000HUA, Argentina; 3 Departamento de Diagnóstico Molecular, LACE Laboratorios, Córdoba X5000JJS, Argentina; portela.nestord@gmail.com; 4 División Microbiología, Laboratorio General y Especial, Hospital Rawson, Córdoba X5000, Argentina; andres.barnes@unc.edu.ar (A.B.); sguadalupec@hotmail.com (S.G.C.); davornm@gmail.com (D.M.); 5 Servicio de Laboratorio General y Especial, Hospital Rawson, Córdoba X5000, Argentina; cesarcollino2013@gmail.com

**Keywords:** microbiota, gut mycobiota, Candida, ITS2, AIDS, Histoplasma

## Abstract

Intestinal dysbiosis is common in people living with HIV/AIDS (PLWH), yet fungal communities of the gut microbiota (mycobiota) remain poorly characterized, especially in severely immunosuppressed patients. We analyzed the gut mycobiota of 33 PLWH and 20 healthy controls from a public hospital in central Argentina. Most PLWH presented with severe immunosuppression (<200 CD4^+^ T cells/μL) and acute or chronic diarrhea, with or without antibiotic exposure or antiretroviral therapy. Fecal DNA was extracted and the ITS2 region was sequenced using next-generation sequencing. Beta-diversity analyses revealed significant segregation between PLWH and controls (PERMANOVA, Adonis: *p* = 0.001, R^2^ = 0.0989). LEfSe analysis identified 17 fungal species enriched in PLWH, predominantly *Candida albicans*, *Candida dubliniensis*, and *Nakaseomyces glabratus*, whereas 31 species were differentially represented in controls, including *Penicillium* spp., *Candida sake*, and *Clavispora lusitaniae*. *Histoplasma capsulatum*, an endemic pathogen in the region, was more prevalent in PLWH and associated with low CD4^+^ T cell counts. Dirichlet multinomial mixture analysis revealed two mycobiotypes: M1, with a balanced fungal composition predominating in controls, and M2, dominated by *Candida* species and present in PLWH. These findings provide novel insights into gut mycobiota alterations in severely immunosuppressed PLWH in Argentina, highlighting *Candida*-driven dysbiosis and the regional relevance of *H. capsulatum*.

## 1. Introduction

In Argentina, approximately 140,000 people are living with HIV/AIDS (PLWH) with nearly 65% receiving care through the public health system. Notably, 44% of new HIV diagnoses occur at a late stage, which favors the development of severe immunosuppression, increases susceptibility to opportunistic infections, and likely predisposes individuals to profound alterations in the composition and diversity of the intestinal microbiota [1,2,3]. In PLWH, intestinal dysbiosis is associated with both local and systemic inflammation, even in patients receiving antiretroviral therapy (ART) [3,4]. Furthermore, colonization resistance against intestinal pathogens depends on specific microbial communities that compete for nutrients and ecological niches [5,6]. Therefore, identifying microbial species and taxa associated with a healthy microbiota, as well as those linked to dysbiosis in PLWH, represents a valuable strategy to guide interventions aimed at restoring or optimizing microbiota composition [7,8].

While most studies have focused on the bacterial component of the microbiota, the fungal counterpart, the gut mycobiota, remains comparatively understudied. Although fungi constitute only a small fraction of the intestinal ecosystem (0.01–0.1%), they play essential roles in intestinal physiology and immune modulation. Alterations in the gut mycobiota have been associated with metabolic, oncological, neurological, inflammatory, and autoimmune diseases [9,10,11,12,13,14,15,16].

Globally, research on the gut mycobiota in PLWH has largely focused on individuals with mild to moderate immunosuppression who are receiving ART. However, there is scarce data on the intestinal fungal composition in subjects with severe immunosuppression (CD4^+^ T-cell counts < 200 cells/μL), a population at high risk of opportunistic infections and of developing acute or chronic diarrhea [17,18,19]. Moreover, most studies on HIV-associated mycobiota have been conducted in North America, Europe, or Africa, highlighting a significant geographical and clinical gap in Latin America [18,20]. In this regard, some fungal species establish stable commensal relationships with the intestinal mucosa, whereas others are transient and influenced by diet, environment, and geographical context [13,21,22,23,24]. These dynamics contribute to significant differences in fungal composition among distinct populations, underscoring the importance of characterizing region-specific microbial profiles.

In this study, we characterized the gut mycobiota of a cohort of HIV-AIDS patients from Argentina, most of whom presented with CD4^+^ T-cell counts below 200 cells/µL, and compared it with that of healthy control individuals. Fungal communities were profiled using high-throughput sequencing of the ITS2 region from fecal DNA, allowing for the assessment of mycobiota diversity and overall community structure. Additionally, we investigated associations between fungal composition and relevant clinical features, including immunological and virological parameters, comorbidities, and therapeutic regimens, with the aim of gaining insights into the relationship between gut mycobiota alterations and disease-related factors in this population.

## 2. Materials and Methods

### 2.1. Subjects

Thirty-three adult patients (men and women, aged 18–61 years) with a confirmed diagnosis of human immunodeficiency virus (HIV) infection were recruited at Rawson Hospital, Córdoba, Argentina. At the time of enrollment, patients were classified according to the presence or absence of diarrhea based on the World Health Organization (WHO) criteria, including acute diarrhea (duration <14 days) and persistent or chronic diarrhea (duration ≥14 days). Thirty participants met the criteria for acute or persistent/chronic diarrhea, while three patients did not report gastrointestinal symptoms at the time of sample collection. Immunological status was assessed at enrollment, and severe immunosuppression was defined as a CD4^+^ T-cell count below 200 cells/µL in peripheral blood. Additionally, twenty healthy individuals without HIV infection, gastrointestinal symptoms, or recent medical treatments including intake of antibiotics, drugs or supplements that could affect gut microbiota in the last 3 months were included as a control group.

### 2.2. Ethics Statement

The study was conducted in accordance with the Declaration of Helsinki and approved by the Comité Institucional de Ética de la Investigación en Salud (CIEIS), Polo Hospitalario del Niño y del Adulto de la Provincia de Córdoba, Argentina (18 May 2020; minutes No. 8; renewed July 2024). Written informed consent was obtained from all participants prior to enrollment. All procedures were performed in accordance with relevant guidelines and regulations.

### 2.3. Fecal Sample Collection

Fecal samples were collected between 2021 and 2023 following the protocols established by the International Human Microbiome Project [24,25]. A sterile and hermetic collection kit was provided to the volunteers for the collection of a fresh stool at home. The stools were frozen at −20 °C, brought to the laboratory within 24 h of collection, and stored at −40 °C until analysis.

### 2.4. DNA Extraction

Fecal samples were handled under sterile conditions inside a laminar flow hood. DNA was isolated using a standardized method involving mechanical lysis with glass beads and the MagMAX™ Microbiome Ultra Nucleic Acid Isolation Kit (Thermo Fisher Scientific, Waltham, MA, USA) ultra-nucleic acid isolation kit on a KingFisher™ Duo Prime Purification System (Thermo Fisher Scientific, Waltham, MA, USA) purification system as previously described [25]. Extracted DNA was stored at −80 °C.

### 2.5. Amplicon Library Preparation of ITS2 rRNA Region, Sequencing, and Fungal Taxonomic Identification

Extracted DNA was amplified using the ProFlex™ PCR System (Thermo Fisher Scientific, Waltham, MA, USA) as previously described [25]. PCR amplification was performed in two steps: first amplification round: Primers ITS1 (5′-TCC GTA GGT GAA CCT GCG G-3′) and ITS4 (5′-TCC TCC GCT TAT TGA TAT GC-3′) were used. The PCR mix (15 μL) contained 12.5 μL of AmpliTaq Gold 360 Master Mix, 1 μL of 360 GC Enhancer (4% *v*/*v*), 0.2 μM of each primer, and 5 μL of template DNA (Thermo Fisher Scientific, Waltham, MA, USA). PCR cycling conditions were: initial denaturation at 95 °C for 10 min; 35 cycles of 95 °C for 30 s, 55 °C for 30 s, and 72 °C for 1 min; followed by a final extension at 72 °C for 7 min. Second amplification round: Primers ITS86 (5′-GTG AAT CAT CGA ATC TTT GAA C-3′) and ITS4 were used. The semi-nested PCR mix contained 3 μL of the first-round product in a 30 μL reaction containing 15 μL of AmpliTaq Gold 360 Master Mix, 1.2 μL of 360 GC Enhancer (4% *v*/*v*), and 0.2 μM of each primer (Thermofisher). Cycling conditions were the same as above. Next, 20 μL of the second-round product was partially digested, barcode adapters were ligated to amplicons (Ion Plus Fragment Library kit, Thermo Fisher Scientific, Waltham, MA, USA), and the product was purified using Agencourt AMPure XP beads (Beckman Coulter, Pasadena, CA, USA) following the manufacturer’s protocol. Extraction blanks and no-template PCR controls were included. Sequencing was performed using the Ion 510TM & Ion 520TM & Ion 530TM Kit—Chef (Thermo Fisher Scientific, Waltham, MA, USA) on the Ion GeneStudio S5 Platform (Thermo Fisher Scientific, Waltham, MA, USA) according to the manufacturer’s instructions. To control for amplification and sequencing, a fungal mock community dataset was generated from mixed DNA of species previously identified by MALDI-TOF: *Candida parapsilosis*, *Candida tropicalis*, *Candida albicans*, *Pichia kudriavzevii (Candida krusei*), *Candida dubliniensis*, *Cryptococcus neoformans*, *Cryptococcus* sp., *Curvularia* sp., *Alternaria* sp., *Fusarium oxysporum*, *Aspergillus* sp., *Saccharomyces cerevisiae*, and *Trichosporum mucoides*.

Sequence quality control, annotation, and taxonomic assignment were performed using the R packages DADA2 v1.22.0, phyloseq v1.38.0, and microbiome v1.16.0 within R software v4.1.219 following the standard protocol from demultiplexed fastq files. The UNITE database formatted for DADA2, version 9.0 (updated October 2022), was used for taxonomic assignment. Sequencing data are accessible in the National Center for Biotechnology Information (NCBI) database under BioProject accession number PRJNA1399939.

### 2.6. Classification of “Mycobiotypes”

Probabilistic modeling of fungal taxonomic data was conducted by grouping mycobiome communities into exclusive metacommunities using the Dirichlet Multinomial Mixture (DMM) model in the R platform. The DMM approach described each community by a vector of taxon probabilities. These vectors were generated based on the optimal number of Dirichlet mixture components selected using the minimum Bayesian Information Criterion (BIC) approximation. The mixture components grouped fungal communities into distinct sample clusters with similar compositions, referred to as “mycobiotypes” in this study. The frequency of mycobiotypes was analyzed across groups presenting different clinical variables (e.g., normal or low CD4^+^ T-cell counts, presence or absence of diarrhea, antibiotic treatment or no treatment, etc.). Chi-square tests were performed to compare mycobiotype frequencies between groups. Subsequently, the abundance of different fungal genera and species associated with each mycobiotype was determined.

### 2.7. Statistical Analysis

Statistical analyses and visualizations were performed using R software version 4.1.2. Normality of variables was assessed with the Shapiro–Wilk test. ANOVA or Kruskal–Wallis tests were applied for simultaneous comparison of more than two variables. Pairwise comparisons were conducted using the Wilcoxon signed-rank test if the Kruskal–Wallis test yielded significant results. Similarly, t-tests were used following significant ANOVA results. *p*-values were adjusted for multiple testing using the Benjamini–Hochberg correction method. Age comparisons between PLWH and controls were performed using the Mann–Whitney U test. For LEfSe analysis, linear discriminant analysis (LDA) scores ≥ 3 and *p*-values < 0.05 were considered significant. Alpha diversity metrics, including observed ASVs, Shannon and Simpson indices, as well as beta diversity metrics (PCoA, and both weighted and unweighted UniFrac), were calculated based on the ASV table representing relative abundances of fungal taxa using the R packages microbiome v1.6.0, phyloseq v1.38.0, and tidyverse v2.0.0. Data visualization was performed with ggplot2 v3.4.0 and ggpubr v0.5.0. A *p*-value < 0.05 was considered statistically significant throughout.

## 3. Results

### 3.1. Demographic and Clinical Characteristics of the Study Participants

The demographic and clinical characteristics of the study participants are summarized in Table 1. Thirty-three (PLWH) and 20 healthy controls were included. There were no statistically significant differences in age between the two groups (*p* = 0.1000). PLWH were predominantly male, and most (n = 29, 87%) presented with severe immunosuppression and detectable HIV viral loads. Diarrhea was frequent among PLWH, including both acute and chronic presentations. Most PLWH required hospitalization, and a substantial proportion were receiving antiretroviral, antibacterial, and/or antifungal treatments at the time of sampling. Oral candidiasis was documented in a subset of PLWH.

### 3.2. Gut Fungal Composition in PLWH Differs from That of HIV-Negative Individuals

**Figure 1:**
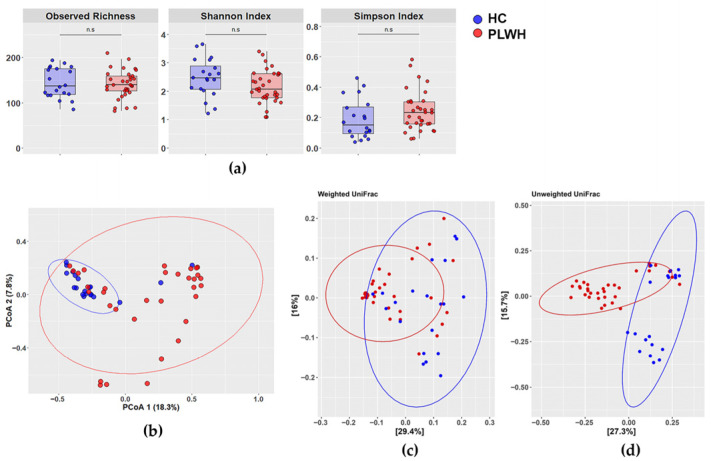
Diversity analyses reveal differences in mycobiota composition between people living with HIV (PLWH) and healthy controls (HC). (**a**) Jitter box plots showing alpha diversity based on observed richness, Shannon index, and Simpson index (Wilcoxon test) comparing PLWH (n = 33) and HC (n = 20). (**b**) Beta diversity analysis: comparison of intestinal mycobiota communities between PLWH and HC using Principal Coordinates Analysis (PCoA). The first two principal coordinates (PCoA1 and PCoA2) are shown (PERMANOVA, Adonis: *p* = 0.001, R^2^ = 0.0989). (**c**,**d**) Beta diversity analysis using UniFrac metrics: (**c**) weighted UniFrac (ANOSIM: *p* = 0.001, R = 0.4357) and (**d**) unweighted UniFrac (*p* = 0.001, R = 0.3406). Ellipses represent the 95% confidence intervals for each group (red: PLWH; blue: HC).

Diversity analysis of the intestinal mycobiota in PLWH and HIV-negative controls showed that alpha diversity (Figure 1a) did not differ significantly between groups across all of the evaluated metrics: observed richness (*p* = 0.6213), Shannon index (*p* = 0.0699), or Simpson index (*p* = 0.105). In contrast, beta diversity assessed by Principal Coordinates Analysis (PCoA) revealed segregation between PLWH and controls, indicating slight differences in mycobiota composition (PERMANOVA, Adonis: *p* = 0.001, R^2^ = 0.0989; Figure 1b). Similar results were obtained using UniFrac distances, which incorporate phylogenetic relationships (Figure 1c,d). Both unweighted UniFrac (presence/absence of taxa) and weighted UniFrac (considering relative abundances) showed significant differences between groups. Notably, the weighted analysis provided greater discriminatory power (Figure 1c; ANOSIM R= 0.436, *p* = 0.001) than the unweighted analysis (Figure 1d; ANOSIM R = 0.341, *p* = 0.001), highlighting that differences in fungal community composition between groups are more pronounced when relative abundances are considered in the analysis.

### 3.3. Distinct Mycobiota Signatures in People Living with HIV: Enrichment of Candida albicans, Candida dubliniensis, Nakaseomyces glabratus, and Histoplasma capsulatum

**Figure 2:**
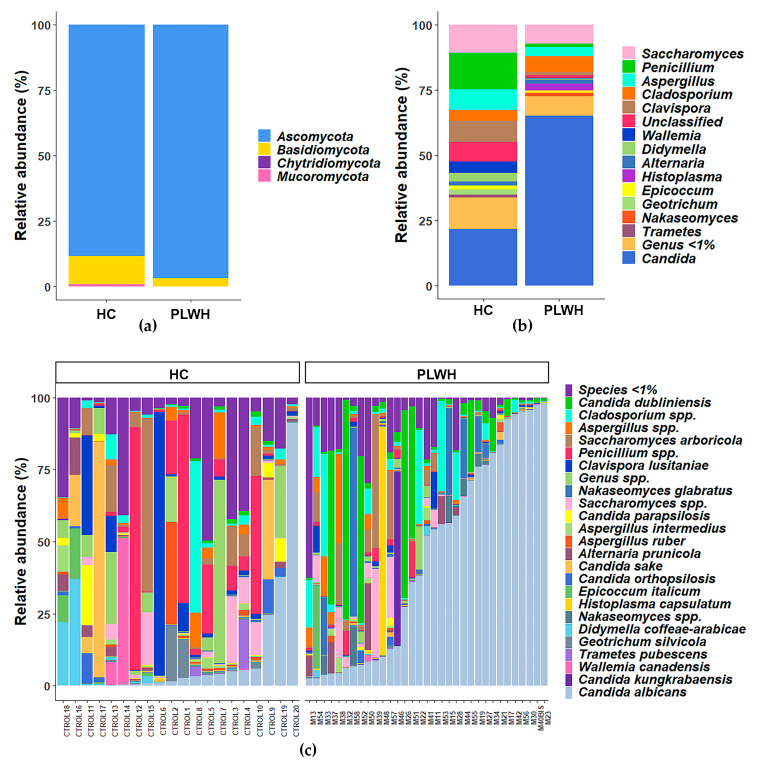
Relative abundance of fungal taxa at the phylum, genus, and species levels in fecal samples. (**a**) Bar plots showing the average relative abundance of the major taxonomic phyla in each group. (**b**) Bar plots representing the average relative abundance of genera with a frequency >1% in each group. (**c**) Individual bar plots illustrating the relative abundance of genera and species in each sample from people living with HIV (PLWH, n = 33) and healthy controls (HC, n = 20). Taxa with a relative abundance <1% of the total were grouped as Genus <1% or Species <1%.

To visualize the overall composition of the gut mycobiota in both groups, we analyzed the assigned taxa (ASVs) with a relative abundance >1%. Figure 2a shows the relative abundance of the fungal phyla detected in each group, while Figure 2b details the distribution of taxa at the genus level. In the control cohort, the gut mycobiota was dominated by Ascomycota, followed by Basidiomycota, with Mucoromycota present at very low levels. In PLWH, the proportion of Ascomycota appeared increased in ITS-based profiles and was primarily associated with *Candida* expansion. Figure 2c shows the relative abundance at the species level for each sample. These results indicate that PLWH exhibit gut mycobiota dysbiosis, with higher relative read fractions of Ascomycota driven by *Candida*-dominated profiles. Notably, this analysis of taxa with a relative abundance >1% further revealed the presence of *Histoplasma capsulatum* in PLWH.

**Figure 3:**
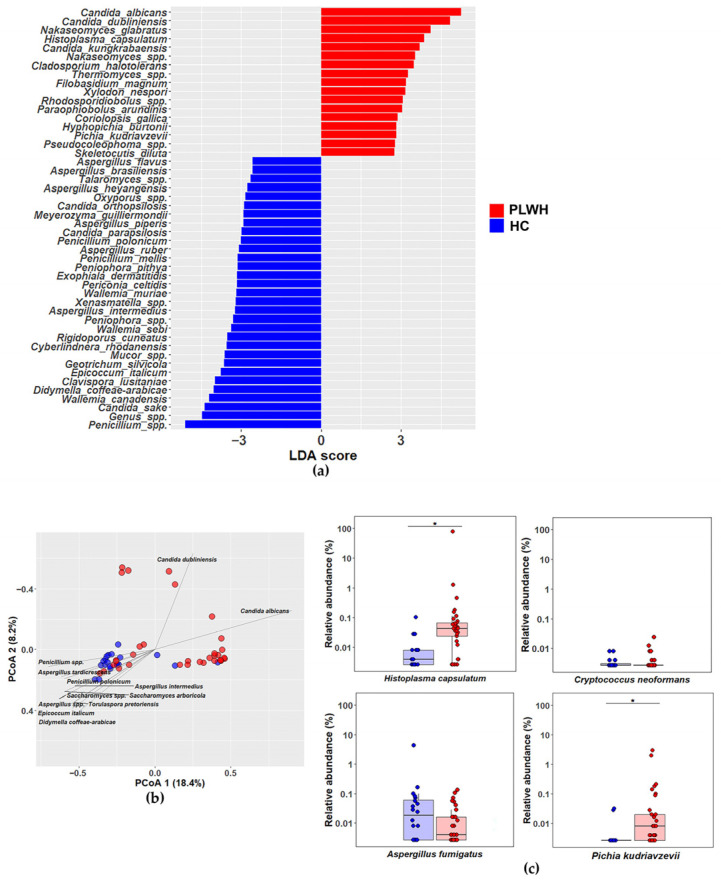
Distinct mycobiota signatures in people living with HIV. (**a**) LEfSe: horizontal bars represent species that differ significantly between groups. LDA scores >3 and *p*-values <0.05 were considered significant. (**b**) Biplot showing vectors that indicate species driving the distribution of taxa in both groups (red: PLWH, blue: healthy controls). (**c**) Relative abundance of common pathogenic fungi in PLWH versus healthy controls. *Pichia kudriavzevii* (formerly *Candida krusei*). * *p* < 0.01, Wilcoxon test.

We performed Linear Discriminant Analysis Effect Size (LEfSe) to determine which taxa statistically differentiate the groups and which have the greatest discriminative effect. This analysis revealed that, among 117 assigned fungal species (ASVs), PLWH showed differential enrichment of 17 taxa, with *Candida albicans*, *Candida dubliniensis*, *Nakaseomyces glabratus* (formerly *Candida glabrata*) being the most discriminatory. LEfSe also described differential enrichment of *Histoplasma capsulatum* and *Pichia kudriavzevii* (formerly *Candida krusei*) in PLWH, two species clinically relevant in immunosuppressed people. In contrast, the healthy controls exhibited higher abundance of 31 species, most prominently *Penicillium* spp., *Candida sake* and *Clavispora lusitaniae* (Figure 3a). Similar results were observed in the statistical comparison of fungal species with a relative abundance >0.1% between PLWH and healthy individuals (Figure S1). Finally, Figure 3b presents a principal coordinates analysis (PCoA) biplot, where vectors represent the species that contribute more to the distribution of the gut mycobiota between groups.

Given that immunosuppressed PLWH are highly susceptible to invasive mycosis caused by *Histoplasma capsulatum*, *Cryptococcus neoformans* and *Aspergillus fumigatus* [26], we further evaluated the relative abundance of these species in the gut mycobiota of PLWH vs. healthy controls. We additionally assessed the relative abundance of *Pichia kudriavzevii*, a clinically relevant yeast due to its intrinsic resistance to azole antifungals [11]. Consistent with its epidemiological relevance and the LEfSe results, *H. capsulatum* was more abundant in the mycobiota of PLWH than in healthy controls. Notably, in at least three healthy individuals, *H. capsulatum* was detected at relative abundances close to 0.1%, comparable to those of other filamentous fungi. Similarly, PLWH exhibited a significant increase in the abundance of *P. kudriavzevii* compared with healthy individuals. By contrast, *C. neoformans* showed very low relative abundance (<0.01%) with no significant differences between groups, and *A. fumigatus* also displayed no group-specific differences (Figure 3c). These data strongly suggest that *Histoplasma capsulatum* is a relevant component of the human gut mycobiota in Argentina, consistent with its status as an endemic pathogenic fungus in the central region of the country and a major cause of disseminated mycoses in immunosuppressed PLWH.

### 3.4. The Gut Mycobiota Clusters into Two Mycobiotypes, M1 and M2, with M1 Predominating in Healthy Controls

**Figure 4:**
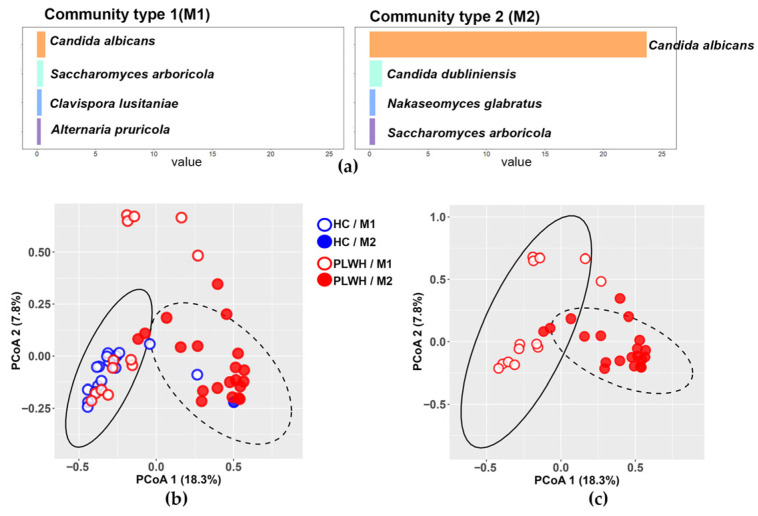
Stratification of the gut mycobiota into fungal community types (mycobiotypes). (**a**) Mycobiotypes were generated using Dirichlet Multinomial Mixture (DMM) modeling. Bars represent proportional values, and the genera and species that characterize each mycobiotype (M1 or M2) are indicated. (**b**) Beta diversity according to the assigned mycobiotype in the total study population (PERMANOVA, Adonis *p* = 0.013, R^2^ = 0.0438) and (**c**) in the population of PLWH (PERMANOVA, Adonis *p* = 0.743, R^2^ = 0.0208). HC: Healthy controls (n = 20); PLWH: People living with HIV (n = 33). HC/M1: HC with mycobiotype 1 (n = 19, blue open circles); HC/M2: HC with mycobiotype 2 (n = 1, blue filled circle); PLWH/M1: PLWH with mycobiotype 1 (n = 13, red open circles); PLWH/M2: PLWH with mycobiotype 2 (n = 20, red filled circles). Ellipses represent the 95% confidence intervals for each group (solid: M1; dashed: M2).

Dirichlet Multinomial Mixture (DMM) analysis identified two distinct mycobiotypes in the fungal communities of participants from both groups: mycobiotype 1 (M1), characterized by balanced abundances of *Candida albicans*, *Saccharomyces arboricola*, *Clavispora lusitaniae*, and *Alternaria pruricola*; and mycobiotype 2 (M2), defined by high abundance of *Candida albicans*, followed by *Candida dubliniensis*, *Nakaseomyces glabratus*, and *Saccharomyces arboricola* (Figure 4a). Beta diversity analysis of all participants, stratified by mycobiotype, revealed slight differentiation in community composition between M1 and M2 (PERMANOVA, Adonis: R^2^ = 0.0438, *p* = 0.013; Figure 4b). The healthy control group predominantly exhibited M1 (19/20 individuals; blue open circles), with only one displaying M2 (blue filled circle). In contrast, PLWH were distributed across both mycobiotypes with 13 individuals exhibiting M1 (red open circles), and 20 M2 (red filled circles) (Figure 4b,c). Notably, among the 33 PLWH, the four individuals with CD4^+^ T-cell counts above 200 cells/µL all displayed an M1 mycobiotype (Figure S2).

We further analyzed clinical variables associated with M1/M2 distribution in PLWH using PCoA, stratified by diarrhea type, recent antibiotic therapy, antifungal treatment, ART status, oral candidiasis, and hospitalization status (outpatient, general ward or ICU). Although M2 tended to be more prevalent among patients with chronic diarrhea, antibiotic therapy, absence of ART or antifungal treatment, oral candidiasis and ICU hospitalization, the differences were not statistically significant (Figure S3).

### 3.5. Impact of Diarrhea and Antibiotic Treatment on the Relative Abundance of Dominant Fungal Species in PLWH

**Figure 5:**
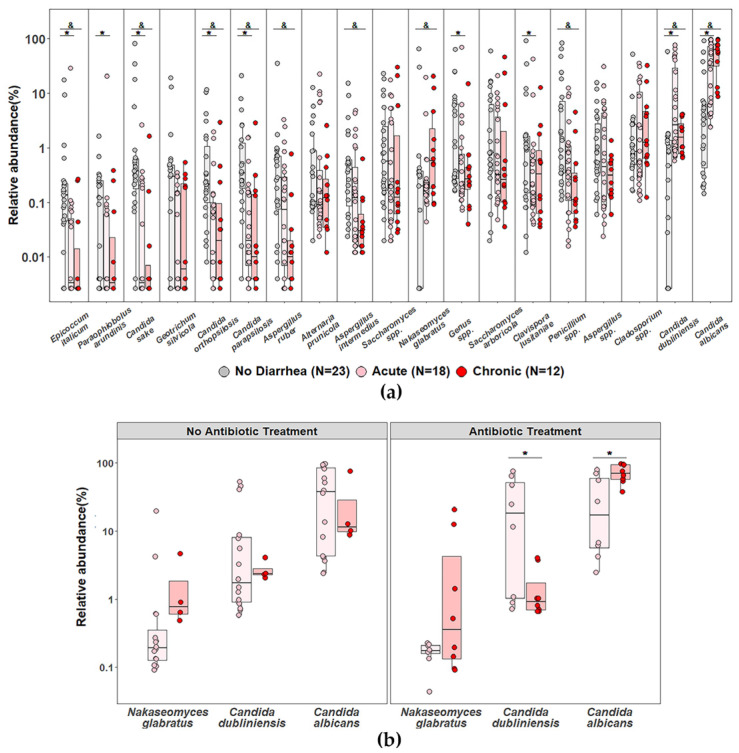
Impact of diarrhea type and antibiotic treatment on the abundance of dominant gut fungi in PLWH. (**a**) Jitter boxplots showing the relative abundances of species present at >0.1%. Light gray bars: individuals without diarrhea (20 controls and 3 PLWH). Pink bars: PLWH with acute diarrhea (<14 days) and red bars: PLWH with chronic diarrhea (>14 days) according to WHO definitions. * *p* < 0.05, acute diarrhea vs. no diarrhea, & *p* < 0.05, chronic diarrhea vs. no diarrhea (pairwise Wilcoxon test after Kruskal–Wallis test). (**b**) Relative abundances of *N. glabratus* (formerly Candida glabrata), *C. dubliniensis*, and *C. albicans* in PLWH with acute (pink) or chronic (red) diarrhea, with or without antibiotic treatment. * *p* < 0.05 (pairwise Wilcoxon test after Kruskal–Wallis test).

Given that most PLWH presented with acute or chronic diarrhea at the time of sampling, we performed an exploratory analysis of the fungal species with relative abundance > 0.1% considering individuals with or without diarrhea (30 PLWH and 20 controls plus 3 PLWH, respectively), as well as under acute or chronic conditions. We consistently observed increased relative abundance of *Candida albicans*, *Candida dubliniensis*, and *Nakaseomyces glabratus* in participants with diarrhea compared with those without diarrhea (*p* < 0.05; pairwise Wilcoxon test after Kruskal–Wallis; Figure 5a). When we evaluated the abundance of these species considering the combination of acute or chronic diarrhea and antibiotic treatment, we found that PLWH with the highest relative abundance of *Candida albicans* were those with chronic diarrhea receiving antibiotic therapy (Figure 5b). In contrast, no statistically significant differences were observed when other clinical variables, such as CD4^+^ T-cell counts, HIV viral load, hospitalization status, presence of oral candidiasis, or antifungal or antiretroviral treatments, were considered.

We also assessed the relative abundance of *Histoplasma capsulatum*; however, no significant differences were detected according to diarrhea status, hospitalization, oral candidiasis, or antiretroviral, antibiotic, or antifungal treatment.

**Figure 6:**
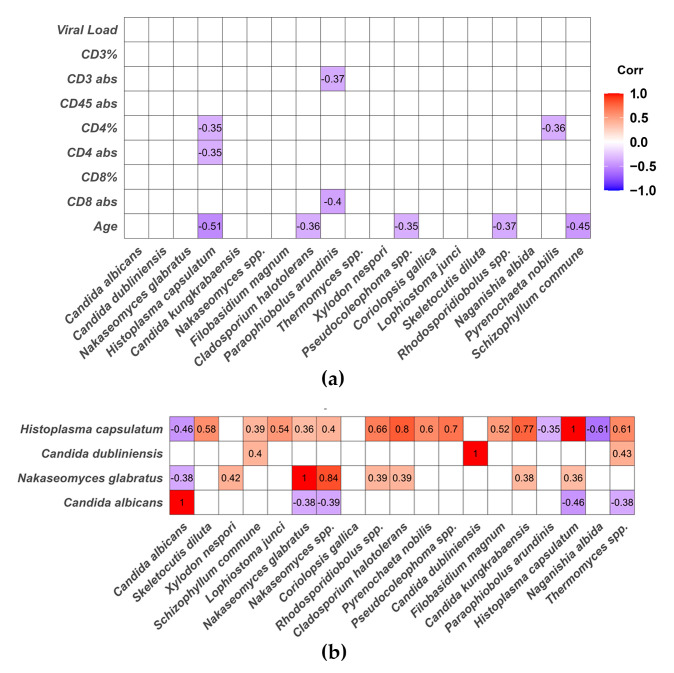
Heatmaps of Spearman correlations between clinical variables and intestinal fungal species abundance. (**a**) Correlations among continuous clinical parameters and the relative abundance of major fungal species in PLWH, and (**b**) correlations fungal species. Colors indicate the strength and direction of the correlation: intense red represents strong positive correlations, blue represents negative correlations, and white indicates no correlation. Only correlations with absolute *p* values ≥ 0.3 are shown.

Furthermore, Spearman correlation analyses among continuous clinical parameters and the relative abundance of the main fungal species differentially detected in PLWH showed that the relative abundance of *Histoplasma capsulatum* correlated negatively with both the absolute count and the percentage of CD4^+^ T cells (rho = –0.35, *p* = 0.045 and rho = –0.35, *p* = 0.043, respectively), as well as with age (rho = –0.51, *p* = 0.003) (Figure 6a). Notably, *Candida albicans* did not show statistically significant correlations with peripheral blood lymphocyte counts, viral load, or patient age. In addition, *Pyrenochaeta nobilis* and *Paraophiobolus arundinis* exhibited negative correlations with lymphocyte counts. Correlation analysis between fungal species revealed that *Candida albicans* was negatively correlated with *Histoplasma capsulatum* (rho = −0.46, *p* = 0.007), *Nakaseomyces glabratus* (rho = −0.38, *p* = 0.027) and *Thermomyces* spp. (rho = −0.38, *p* = 0.028) (Figure 6b).

## 4. Discussion

Generally, microbiota research in PLWH has predominantly focused on bacterial communities, mainly in cohorts with mild to moderate immunosuppression from Europe, the United States, and Africa [8,18,27]. Consequently, data on the intestinal microbiota of severely immunocompromised PLWH, particularly from Latin America, remain scarce [28,29]. Our study addresses this important geographical and clinical gap by providing a comprehensive characterization of gut fungal dysbiosis in a cohort of severely immunosuppressed PLWH from central Argentina.

The global burden of fungal diseases continues to increase, driven by factors such as climate change, the growing population of individuals with immunosuppressive conditions, and the rise in antimicrobial resistance. Fungal infections remain a major cause of morbidity and mortality among individuals with advanced HIV disease. Intestinal colonization or dysbiosis dominated by potentially pathogenic or antifungal-resistant fungi may increase the risk of both local and systemic infections. In addition, alterations in gut microbiota composition can promote chronic inflammation in PLWH [16,26] and fungal dysbiosis may influence disease progression and therapeutic responses [30]. In this study, we characterized the diversity and composition of the gut mycobiota using NGS-based analysis of the ITS2 region from fecal samples of PLWH with severe immunosuppression, most of whom had CD4^+^ T-cell counts below 200 cells/µL and presented with acute or chronic diarrhea. This cohort is clinically relevant, as late HIV diagnosis exceeds 44% in Argentina, increasing susceptibility to intestinal mucosal damage and co-infections with opportunistic pathogens [1,26].

Beta-diversity analyses demonstrated that PLWH exhibit a gut fungal composition significantly distinct from that of healthy controls, particularly when relative abundances of taxa were considered, suggesting that specific fungal species may expand and outcompete others in this patient population. Among the 117 species identified by NGS, LEfSe analyses showed differential representation of 17 species in PLWH and 31 species in healthy controls.

Among the 117 species identified by NGS, LEfSe analyses revealed that 17 species were enriched in PLWH, whereas 31 were more abundant in healthy controls. The mycobiota of PLWH was characterized by marked dysbiosis dominated by *Candida albicans* and *Candida dubliniensis*, and, to a lesser extent, by clinically relevant yeasts such as *Nakaseomyces glabratus* (formerly *Candida glabrata*), *Pichia kudriavzevii* (formerly *Candida krusei*), and the endemic pathogen *Histoplasma capsulatum*.

The fungal composition observed in HIV-negative controls is consistent with previous reports [10,24,31], being dominated by Ascomycota (>80%), followed by Basidiomycota and a low representation of Mucoromycota. Although fungal communities are known to be more variable than bacterial communities [24,31] a conserved “fungal core” has been described in healthy adults [24]. This core closely matched our control cohort, characterized by genera such as *Saccharomyces*, *Penicillium*, *Aspergillus*, *Cladosporium*, *Clavispora*, *Wallemia*, *Alternaria*, *Epicoccum*, *Geotrichum*, and *Candida*. Yeasts of the genus *Candida*, particularly *Candida albicans*, are stable colonizers of the intestinal, oral, and vaginal mucosa [11]. Recent evidence indicates that *Candida* and *Saccharomyces* species can penetrate deeper into the intestinal mucosa, where they may influence immune, metabolic, and even behavioral host responses under homeostatic conditions [13]. In contrast, filamentous fungi such as *Penicillium*, *Aspergillus*, *Alternaria*, and *Cladosporium* are largely environmental or food-borne and appear to transiently colonize the intestinal lumen with limited host interaction [9,21,31].

The fungal profile observed in PLWH aligns with well-established evidence indicating that intestinal dysbiosis in this population is associated with an increased abundance of *Candida* spp., which has been associated with inflammation, mucosal barrier dysfunction, and an increased risk of systemic infection.

*Candida albicans* was the predominant species, consistent with its strong adhesive capacity, biofilm formation, and yeast-to-hypha transition [21]. In PLWH, *Candida albicans* remains the principal etiological agent of oropharyngeal and esophageal candidiasis [31]. Notably, *Candida dubliniensis*, a closely related species with important genetic and phenotypic differences, was also highly abundant. Although generally susceptible to azoles and echinocandins, *Candida dubliniensis* can rapidly develop fluconazole resistance under antifungal pressure [32]. While frequently isolated from the oral cavity of HIV-infected individuals, its presence in the gut mycobiota has rarely been reported [33]. Our data indicate that *Candida dubliniensis* is a relevant and previously underrecognized component of the gut mycobiota in severely immunosuppressed PLWH.

Increased relative abundance of *Nakaseomyces glabratus* and, to a lesser extent, *Pichia kudriavzevii* was also observed. Both species are clinically relevant due to their intrinsic or acquired resistance to azole antifungals and their capacity to adapt to environmental stress, form biofilms, and express virulence factors [34]. Their enrichment in PLWH suggests that severe immunosuppression-associated gut fungal dysbiosis may favor the expansion of clinically relevant yeasts. Nevertheless, these findings do not allow us to infer direct selection of antifungal-resistant fungi, and this hypothesis should be addressed in future longitudinal and culture-based studies. These species are also included in the WHO fungal priority pathogens list [35], underscoring their global clinical relevance in the context of antifungal resistance.

Other species differentially detected in PLWH by LEfSe may share common ecological or biological traits. For instance, *Filobasidium magnum* and *Rhodosporidiobolus* spp. are yeast-forming basidiomycetes capable of colonizing skin or mucosal surfaces. *Rhodosporidiobolus fluvialis* has been reported to exhibit high resistance to fluconazole and caspofungin [36]. *Candida kungkrabaensis* and *Hyphopichia burtonii* are ascomycetous yeasts that may behave as transient commensals or even opportunistic pathogens under immunosuppressive conditions [37]. Finally, several species display adaptations that may favor their survival in the intestinal environment, including thermotolerance (e.g., *Thermomyces* spp. and *Pichia kudriavzevii*) and halotolerance or osmotolerance (e.g., *Cladosporium halotolerans* and *Hyphopichia burtonii*).

Consistent with previous studies, *Candida* spp., particularly *Candida albicans*, were more prevalent in PLWH with diarrhea and recent antibiotic exposure [38]. In contrast to other reports [39], we did not observe an increased abundance of *Candida parapsilosis* or *Candida tropicalis*, which may reflect the specific clinical characteristics of our cohort, including severe immunosuppression and active diarrhea, likely favoring the prevalence of species better adapted to the intestinal niche.

Remarkably, our study detected *Histoplasma capsulatum* sequences in the gut mycobiota, with a higher relative abundance in PLWH compared with healthy controls. To our knowledge, this is the first NGS-based study reporting Histoplasma DNA detection in the intestinal mycobiota. While the highest relative abundances were observed in individuals with disseminated histoplasmosis, the detection of this fungus in multiple PLWH and even in some healthy individuals suggests a possible transient or subclinical presence within the gastrointestinal tract. Importantly, *Histoplasma capsulatum* is also classified within the WHO priority fungal pathogens, reinforcing its clinical relevance, particularly in endemic regions such as Latin America. Whether the gut may act as a reservoir or reflect systemic dissemination remains speculative and warrants further investigation.

Dirichlet Multinomial Mixture analysis identified two distinct mycobiotypes: one predominating in healthy individuals and another characterized by high *Candida albicans* abundance. Although both mycobiotypes were present in PLWH, no significant associations with clinical or immunological parameters were observed, likely due to sample size limitations or the complex nature of host–fungal interactions. In this context, it is important to note that, similar to healthy controls, the four individuals in the PLWH group with moderate immunosuppression or no immunosuppression exhibited a balanced M1 mycobiotype.

Spearman correlation analysis demonstrated a significant negative correlation between CD4^+^ T-cell counts and the relative abundance of *Histoplasma capsulatum*, consistent with the well-established role of CD4^+^ T cells in the control of intracellular fungal pathogens. Additionally, a negative correlation between *Candida albicans* and *Nakaseomyces glabratus* or *Histoplasma capsulatum* was detected, suggesting that, although these species are more abundant in PLWH compared to healthy individuals, they do not necessarily increase simultaneously within the same subject. These findings may reflect niche-specific fungal expansion and potential interspecies competition within the gut ecosystem under conditions of severe immunosuppression.

Overall, this study represents the first comprehensive characterization of gut fungal dysbiosis in severely immunosuppressed PLWH with diarrhea in a public hospital setting in central Argentina. Interestingly, the highest relative abundance of *Candida albicans* was observed in PLWH with chronic diarrhea receiving antibiotic therapy, suggesting a potential interaction between bacterial microbiota disruption and fungal expansion. These observations further support the concept that fungal dysbiosis in PLWH is shaped by the interplay between host immune status, antimicrobial exposure, and gastrointestinal conditions, reinforcing the need for integrative approaches to understand its clinical implications.

Strengths and limitations:

From a translational perspective, the characterization of gut fungal communities in a Latin American cohort of severely immunosuppressed PLWH provides clinically relevant context for interpreting fungal colonization and dysbiosis patterns in routine care, particularly in populations with a substantial burden of opportunistic mycoses.

The predominance of Candida-driven mycobiota profiles (M2) and the detection of *Histoplasma capsulatum* sequences in this cohort highlight the need to consider fungal community structure as a potential marker of mucosal imbalance and infection risk, particularly in patients with advanced immunosuppression and gastrointestinal symptoms.

Notably, several of the fungal taxa enriched in our cohort, including *Candida albicans, Nakaseomyces glabratus, Pichia kudriavzevii, and Histoplasma capsulatum*, are recognized as priority fungal pathogens by the World Health Organization (WHO), highlighting the potential clinical relevance of the observed mycobiota alterations in the context of global public health and antifungal resistance.

Although causality cannot be inferred, these findings suggest that integrating mycobiota profiling with clinical and immunological parameters may contribute to improved risk stratification, support earlier suspicion of opportunistic mycoses in endemic settings, and inform more targeted use of antifungal and antibiotic therapies. Further longitudinal and functional studies will be required to determine whether specific fungal signatures can serve as actionable biomarkers in clinical practice.

Due to the clinical characteristics of the cohort, it is not possible to draw conclusions regarding intestinal fungal dysbiosis associated exclusively with HIV infection. In addition, the cross-sectional design limits causal inference, and the relatively small sample size may have reduced the power to detect associations between fungal community structures and clinical or immunological variables. Furthermore, the lack of functional or culture-based validation limits the ability to determine the viability and clinical significance of the detected fungal taxa. Finally, the amplicon-based approach provides taxonomic but not functional insights, underscoring the need for longitudinal and multiomics studies to better understand the role of gut fungi in advanced HIV disease.

## Figures and Tables

**Table 1:** Demographic data of study participants.

	PLWH (n = 33)	HC (n = 20)
Age (years)		
median (min-max)	42.0 (23–61)	31.5 (25–65)
Sex		
Male/Female (n)	23/10	11/9
CD4^+^ T lymphocytes		
Absolute number/µL: median (min-max)	50 (2–1886)	RR: 410–1590
CD8^+^ T lymphocytes		
Absolute number/µL: median (min-max)	470 (122–3518)	RR: 190–1140
CD3^+^ T lymphocytes		
Absolute number/µL: median (min-max)	559 (135–3693)	RR: 690–2540
Viral Load		
Copies/mL: median (min-max)	173,000 (0–10,000,000)	Undetectable
Diarrhea *		
Chronic/Acute/No diarrhea (n)	12/18/3	0/0/20
Hospitalization		
Outpatient/Inpatient/ICU (n)	8/20/5	20/0/0
Antiretroviral treatment (ART)		
Yes/No (n)	13/20	0/20
Antibacterial treatment (ATB)		
Yes/No (n)	17/16	0/20
Antifungal treatment (ATF)		
Yes/No (n)	9/24	0/20
Oral Candidiasis		
Yes/No (n)	8/25	0/20

PLWH: people living with HIV (n = 33). HC: Healthy controls: healthy HIV-negative individuals (n = 20). n: Number of participants. RR: reference range. * Diarrhea type classification according to the World Health Organization (WHO): persistent/chronic > 14 days; acute: <14 days. ICU: Intensive care unit.

## Data Availability

Sequencing data are accessible in the National Center for Biotechnology Information (NCBI) database under BioProject accession number: PRJNA1399939.

## Supplementary Materials

The following supporting information can be downloaded at: https://www.mdpi.com/article/10.3390/jof12050306/s1, Figure S1: Distinct mycobiota signatures in people living with HIV. Relative abundance of species with >0.1% abundance across groups. Groups were compared using the Wilcoxon test, with *p* < 0.05 after Benjamini–Hochberg adjustment. Blue bars indicate healthy controls, and red bars indicate PLWH. Figure S2: Distribution of CD4^+^ T-cell counts in PLWH. Boxplot of peripheral blood CD4^+^ T-cell counts in PLWH (n = 33) stratified by mycobiotype: M1 (open red circles, n = 13) and M2 (filled red circles, n = 20). Boxes represent the interquartile range with median; whiskers indicate the range. Figure S3: Associations between mycobiotypes and categorical clinical variables in PLWH. (A) Beta-diversity analysis of M1 and M2 in PLWH and their relationship with comorbidities, treatments, and hospitalization. (B) Bars represent the percentage of PLWH with M1 and M2 in each condition. None of these associations is statistically significant (chi-square test). PLWH: People living with HIV-AIDS, n = 33.

## Abbreviations

The following abbreviations are used in this manuscript:

PLWH	People living with Human Immunodeficiency Virus
LEFSe	Linear Discriminant Analysis Effect Size
DMM analysis	Dirichlet multinomial mixture analysis
ART	antiretroviral therapy
