# Supplementary material for: Gut Mycobiota Dysbiosis in People Living with HIV/AIDS: Insights from an Argentine Cohort with Severe Immunosuppression
Source: J Fungi (Basel). 2026 Apr 23;12(5):306. doi: 10.3390/jof12050306 (PMC13207828; doi:10.3390/jof12050306)
Supplement: Supplementary file 1 [file jof-12-00306-s001.zip › jof-4209320-supplementary.pdf]

Supplementary Data

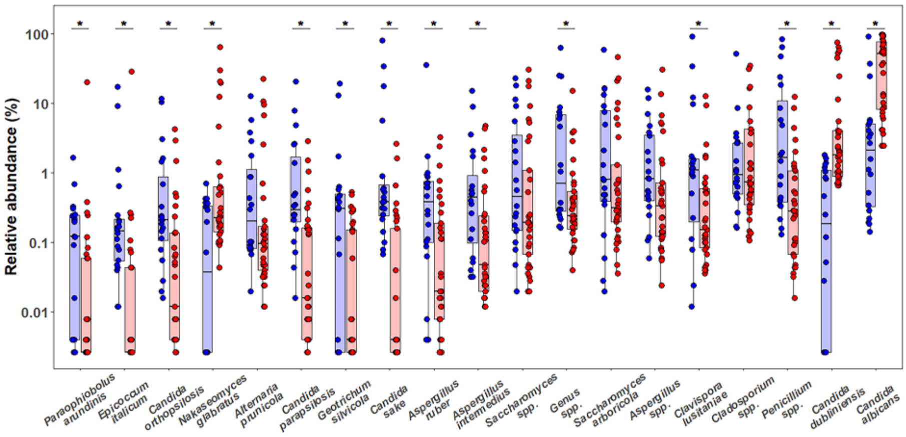

Supplementary Figure S1. Distinct mycobiota signatures in people living with HIV. Relative abundance of species with > 0.1% abundance across groups. Groups were compared using the Wilcoxon test, with $p < 0.05$ after Benjamini-Hochberg adjustment. Blue bars indicate healthy controls, and red bars indicate PLWH.

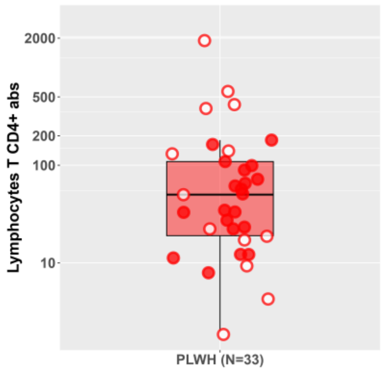

Supplementary Figure S2. Distribution of CD4$^+$ T-cell counts in PLWH. Boxplot of peripheral blood CD4$^+$ T-cell counts in PLWH (n = 33) stratified by mycobiotype: M1 (open red circles, n = 13) and M2 (filled red circles, n = 20). Boxes represent the interquartile range with median; whiskers indicate the range.

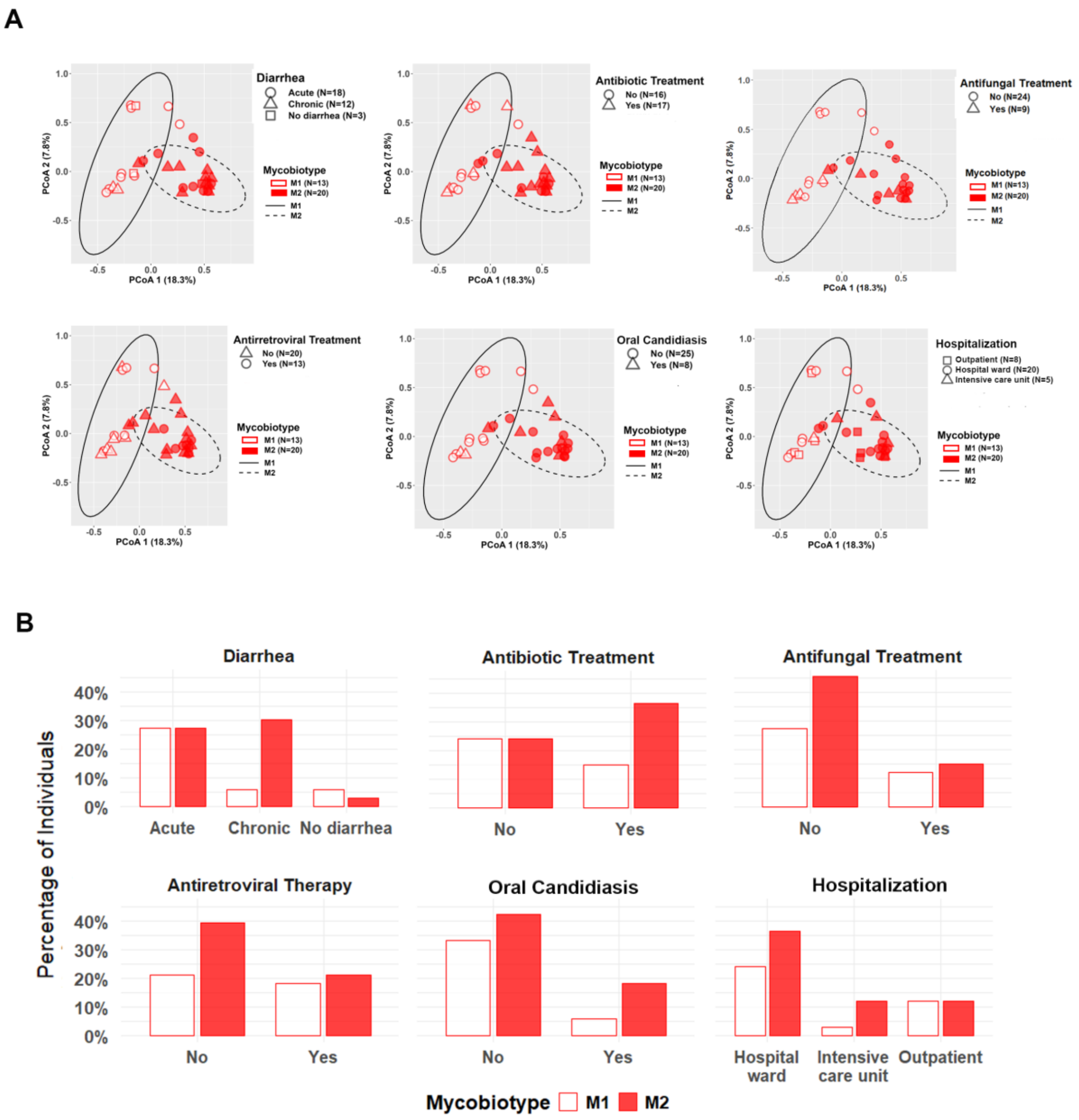

Supplementary Figure S3. Associations between mycobiotypes and categorical clinical variables in PLWH. (A) Beta-diversity analysis of M1 and M2 in PLWH and their relationship with comorbidities, treatments, and hospitalization. (B) Bars represent the percentage of PLWH with M1 and M2 in each condition. None of these associations is statistically significant (chi-square test). PLWH: People living with HIV-AIDS, n= 33.
